# A Long-Term Prospective Evaluation of the Predictive Value of Molecular Markers in Serum and Cerebrospinal Fluid in Patients in the Early Stage of Multiple Sclerosis

**DOI:** 10.3390/ijms27073155

**Published:** 2026-03-31

**Authors:** Mariola Świderek-Matysiak, Magdalena Oset, Karolina Pendrasik, Dominika Świerczewska, Małgorzata Domowicz, Magdalena Namiecińska, Mariusz Stasiołek

**Affiliations:** 1 Department of Neurology, Medical University of Lodz, Kosciuszki Street 4, 90-419 Lodz, Poland; magdalena.oset@umed.lodz.pl (M.O.); karolina.pendrasik@umed.lodz.pl (K.P.); dominika.swierczewska@umed.lodz.pl (D.Ś.); malgorzata.domowicz@umed.lodz.pl (M.D.); mariusz.stasiolek@umed.lodz.pl (M.S.); 2 Department of Immunogenetics, Medical University of Lodz, Kosciuszki Street 4, 90-419 Lodz, Poland; magdalena.namiecinska@umed.lodz.pl

**Keywords:** multiple sclerosis, prognosis, biomarkers, neurofilaments, interleukin 6

## Abstract

Various molecular biomarkers have been suggested as a method to improve the predictive value of prognosis in multiple sclerosis (MS). The characterization of such biomarkers would greatly enhance individual patient management. The aim of this study was to conduct a long-term, prospective evaluation of the prognostic value of molecular biomarkers in serum and cerebrospinal fluid (CSF) in patients in the early stages of MS, before the first treatment initiation. The study included a total of 121 MS patients. Serum and CSF were obtained during diagnostic process. Concentrations of IFN **γ**, IL-6, CHI3L1, osteopontin, CXCL13, GFAP and neurofilament light chains (NfLs) were analyzed. The mean time of the observation of clinical and radiological MS activity was 60 months after start of MS treatment. Higher serum concentrations of NfLs (Z = 2.28; *p* = 0.02) and CXCL13 (Z = 2.14; *p* = 0.03) correlated with need to change the first MS therapy (88% due to ineffectiveness and 12% due to adverse events). Higher NfLs concentrations in the CSF specifically correlated with the occurrence of radiological activity (Z = 2.02; *p* = 0.04). Increased NfLs and IL-6 concentrations in the CSF correlated with disability progression assessed with the EDSS (Z = 2.81; Z = 2.87; *p* = 0.004; *p* = 0.003, respectively), as well as with clinical and/or radiological disease activity (Z = 2.80; Z = 2.43; *p* = 0.004; *p* = 0.014, respectively). High levels of NfLs and Il6 in the CSF of MS patients assessed before the therapy may be an indication to use a highly effective therapy as the first treatment for MS.

## 1. Introduction

Multiple sclerosis (MS) is a chronic demyelinating disease of the central nervous system (CNS) with inflammation and neurodegeneration underlying the clinical symptoms [1]. Although in the great majority of patients (ca 85%) disease begins with the relapsing–remitting phenotype (RRMS), often referred to as an “inflammatory stage”, the current understanding of MS biology indicates that neurodegenerative processes are present from the very beginning and even precede the first clinical symptoms during the prodromal phase of MS [2]. Accordingly, magnetic resonance imaging (MRI) scans reveal that brain volume loss occurs from the onset, and this damage progresses similarly to later stages. Early intervention is crucial, as the brain repair mechanisms are limited and become less effective over time [3]. A growing body of evidence supports the clinical advantages of an early initiation of disease modifying treatment (DMT) in RRMS, making it a key recommendation in modern treatment guidelines [4,5,6]. However, the pathophysiology of MS changes with time and numerous studies suggest that early identification of patients at risk of transitioning to secondary progressive MS (SPMS) allows for appropriate planning and/or adjustment of therapeutic strategies [4,7]. In 2003, Confavreux et al. highlighted key clinical factors that are useful in predicting MS progression, such as patient demographics (age and gender), initial symptoms, recovery after the first episode, time to a second episode, and relapse frequency within five years. Various molecular biomarkers have been suggested as a method to improve the predictive value of clinical and radiological parameters in MS [8]. However, identification of biomarkers that can effectively detect the subtle, ongoing processes involved in the progression of MS is difficult and demands further research [2,9].

Importantly, the potential clinical application underlines the need to develop biomarkers that are easily and reliably detectable in body fluids such as cerebrospinal fluid (CSF) and, optimally, in peripheral blood (serum/plasma), which would allow for implementation in the monitoring of disease activity and therapy efficacy. The characterization of such biomarkers would greatly enhance patient management, and lead to more personalized and timely interventions in clinical practice [10].

CSF-specific oligoclonal bands (OCBs), have been for many years considered as the gold standard for detection of intrathecal inflammation in MS. Numerous other inflammatory biomarkers, non-specific for MS, like IL-6, interferon-gamma, CXCL13 and CHI3L1, provide key insights into MS disease activity and immune mechanisms, aiding in the differentiation of disease stages and prediction of treatment responses [10,11,12,13]. Neuronal damage and degeneration, on the other hand, are indicated by the increase of neurofilament light chains (NfLs) and GFAP (glial fibrillary acidic protein) both in CSF and serum. NfLs are released during axonal damage, while GFAP reflects astrocyte activation, both serving as key biomarkers for disease progression [14]. In the case of NfLs, correlation with relapse and MRI activity was also documented [15].

The aim of this study was to conduct a long-term, prospective evaluation of the prognostic and predictive value of molecular biomarkers in peripheral blood and CSF in patients in the early stages of MS, in the time after the MS diagnosis but before the first DMT initiation.

## 2. Results

### 2.1. Demographic Patients Characteristic and MS Disease Activity

Demographic and medical data of all the participants were collected prospectively in a local medical database. The mean time of observation was 60 months (median 50 months) after start of MS treatment. The study included a total of 121 patients, 81 (67.5%) women and 40 (32.5%) men, with a mean age of 35.26 years during diagnosis. The mean time from the first signs of MS to diagnosis was 36.1 months and the mean baseline Expanded Disability Status Score (EDSS) was 1.5 points. A total of 95.87% of patients were CSF oligoclonal band-positive. Patients were included in the study as their final RRMS diagnosis was being determined according to the McDonald 2017 criteria. Patients started DMT during the 3 months after MS diagnosis. All medications were used at standard approved doses for MS treatment. The first DMTs in the study group were as follows: dimethyl fumarate (77.1%), glatiramer acetate (7.6%), teriflunomide (6.8%), interferon beta (3.4%), natalizumab (2.5%), fingolimod (1.7%), and cladribine (0.8%). In total 33 patients (27.3%) switched their first DMT during the observation period, among them 88% switched due to therapy ineffectiveness, and four patients switched due to adverse events during therapy. Progression in EDSS score was observed in 19 patients (15.7%) (in eight patients this progression was related to relapses) (mean baseline EDSS 1.5 vs. 1.7 at the end of the observation) and 10 patients (8.3%) had evidence of MS radiological activity (defined as new T2 lesions and/or T1 Gd-enhancing lesions on brain MRI). Of those with radiological activity, six patients developed new T2 lesions on brain MRI, while four had both new T2 lesions and Gd+ enhancing lesions. Among patients with MS activity during the observation period, 24 patients (19.8%) experienced radiological and/or clinical activity. Five patients had their therapy changed due to therapeutic benefits as reported by medical source data, without MS activity seen on an MRI or EDSS assessment. The demographic data are presented in Table 1.

### 2.2. Assessment of Serum and CSF Biomarkers and Correlation with MS Activity

In our study, we assessed concentrations of IFN **γ**, IL-6, CHI3L1, osteopontin, CXCL13, GFAP and NfLs in both the serum and cerebrospinal fluid of patients undergoing a diagnostic workup for multiple sclerosis. The results were then correlated with the course of the disease in the first years and the response to the first MS therapy.

**Figure 1:**
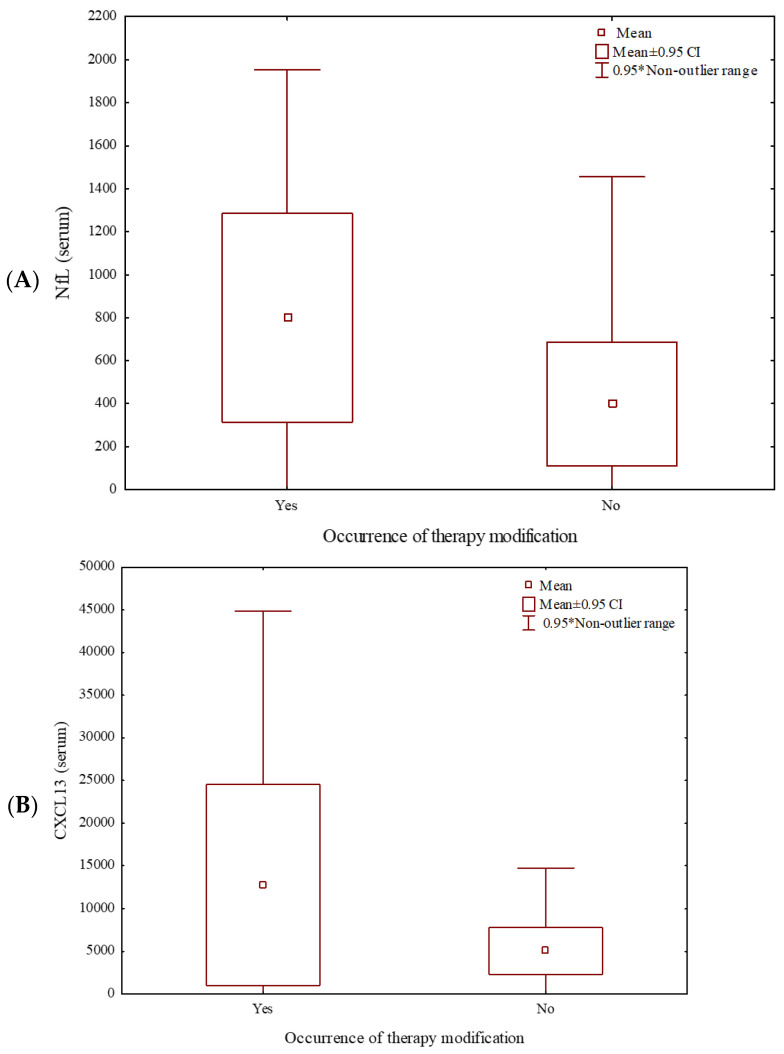
Mean NfLs (**A**) and CXCL13 (**B**) serum concentration correlated with the need for first MS therapy modification (Mann—Whitney U Test: Z = 2.28; *p* = 0.02; Z = 2.14; *p* = 0.03, respectively).

**Figure 2:**
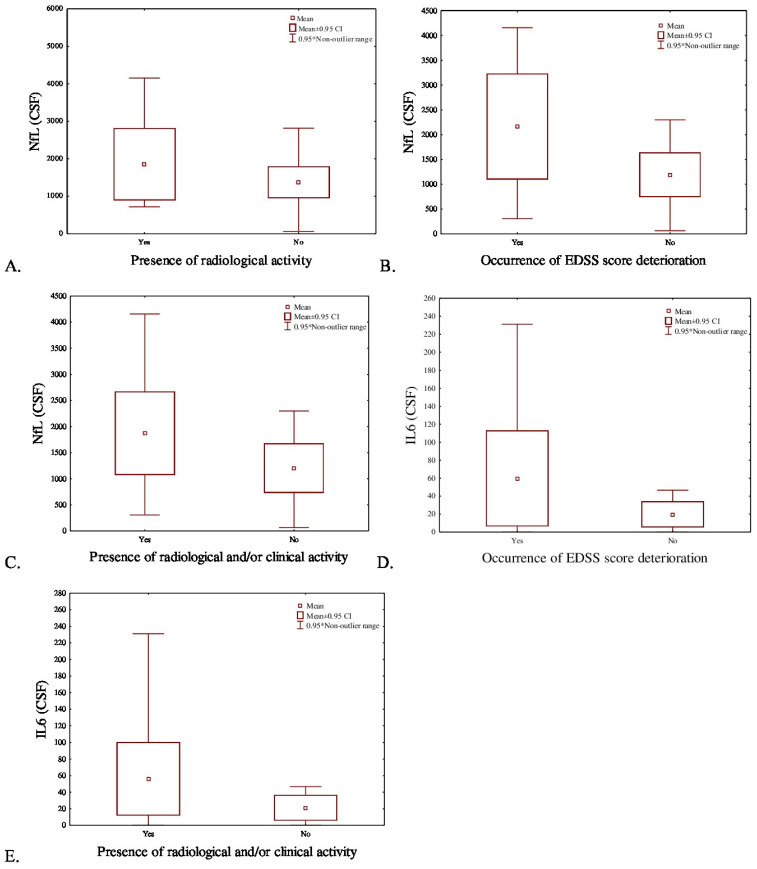
Distribution of mean NfLs concentration in the CSF according to presence of (**A**) radiological activity (Mann–Whitney U Test: Z = 2.02; *p* = 0.04), (**B**) EDSS score deterioration during MS treatment (Z = 2.81, *p* = 0.004) and (**C**) radiological and/or clinical MS activity (Mann–Whitney U Test: Z = 2.80, *p* = 0.004). Distribution of mean IL-6 concentration in the CSF according to the presence of (**D**) EDSS score deterioration (Z = 2.87, *p* = 0.003) and (**E**) radiological and/or clinical activity (Z = 2.43, *p* = 0.014) during MS treatment.

In the linear regression model, the levels of assessed biomarkers were independent of age. On average, higher serum concentrations of NfLs (Mann–Whitney U Test: Z = 2.28; *p* = 0.02) and CXCL13 (Mann–Whitney U Test: Z = 2.14; *p* = 0.03) correlated with the first MS therapy change (88% due to the ineffectiveness and 12% due to adverse events) (Figure 1). No correlation was detected between need for treatment modification and CHI3L1, GFAP, OPN, IL-6, and INF serum levels (Mann–Whitney U Test: Z = 1.29; Z = 0.53; Z = 0.93; Z = 1.74; Z = 0.34; *p* = 0.20; *p* = 0.36; *p* = 0.35; *p* = 0.16; and *p* = 0.58, respectively). None of the evaluated CSF biomarkers showed statistically significant correlations with a need to modify the DMT. Higher NfLs concentrations in the CSF specifically correlated with the occurrence of radiological activity (Mann–Whitney U Test: Z = 2.02; *p* = 0.04) (Figure 2A). There were no other significant correlations between analyzed CSF biomarkers and radiological activity in the CSF (Mann–Whitney U Test: Z = 1.06; Z = −0.99; Z = 0.58; Z = 0.11; Z = 1.71; Z = −0.90; *p* = 0.29; *p* = 0.32; *p* = 0.56; *p* = 0.92; *p* = 0.09; and *p* = 0.37, for CHI3L1, GFAP, CXCL13, OPN, IL-6, and INF, respectively). Similarly, none of the investigated serum molecular biomarkers correlated with radiological activity in our group (Mann–Whitney U Test: Z = −0.74; Z = 1.14; Z = 0.90; Z = 0.70; Z = −0.56; Z = 1.16; Z = −0.36; *p* = 0.46; *p* = 0.25; *p* = 0.37; *p* = 0.48; *p* = 0.58; *p* = 0.24; and *p* = 0.71, for CHI3L1, NfLs, GFAP, CXCL13, OPN, IL-6, and INF, respectively).

Increased NfLs and IL-6 concentrations in the CSF correlated with the disability progression assessed by EDSS (Mann–Whitney U Test: Z = 2.81; Z = 2.87; *p* = 0.004; *p* = 0.003, respectively) (Figure 2B,D), as well as with clinical and/or radiological disease activity (Mann–Whitney U Test: Z = 2.80; Z = 2.43; *p* = 0.004; *p* = 0.014, respectively) (Figure 2C,E). The results are shown in Table 2.

## 3. Discussion

Selecting the most appropriate treatment for MS patients is highly complex. Early therapy with high-efficacy DMT is ideal for MS patients at a high risk of developing active/aggressive MS. The risk of MS activity and progression could be identified at the early MS stage by the presence of predictive biomarkers [8].

Oligoclonal bands (OCBs) are immunoglobulin G (IgG) proteins produced intrathecally by plasma cells. They are detected in the CSF and serum using isoelectric focusing. OCBs are the only validated biological marker in current MS diagnostic criteria [7]. They are a strong predictor of conversion from clinically isolated syndrome (CIS) to MS [15,16]. However, OCBs are not specific to MS and can appear in other central nervous system diseases, for example, neurolupus, sarcoidosis, CNS vasculitis or neuromyelitis optica spectrum disorders [15,17].

In this prospective study, we tried to identify molecular predictors of disease progression at the early stage of RRMS. During long-term observation (mean 60 months; median 50 months), beginning at the time of MS diagnosis and initiation of the therapy, we reported a significant correlation between baseline NfLs and IL6 levels in CSF with the clinical and/or radiological activity of disease during treatment with the first MS drug. The high costs and limited access to serum NfLs measurements with the SIMOA method limit the wider use of NfLs assessment in real-world conditions. For this reason we decided to use ELISA as the most accessible method, which is possible to use in routine clinical practice. NfLs are neuronal cytoskeletal proteins released into CSF and blood during axonal damage, an early process in MS pathology [15]. Levels of NfLs in CSF are considered a strong prognostic marker for assessing disease activity in patients with clinically isolated syndrome (CIS) and RRMS [18]. In addition, serum NfLs have shown potential as a biomarker for evaluating treatment effectiveness in RRMS and as a substitute for MRI in monitoring subclinical disease activity [19]. Furthermore, elevated NfLs levels have been identified as predictors of long-term physical and cognitive impairment following optic neuritis, which often represents the first clinical presentation of central nervous system demyelination. NfLs are also an independent risk factor for conversion to CIS or clinically definite MS in individuals with radiologically isolated syndrome (RIS) [20]. In our study, a strong association between increased NfLs CSF levels at the moment of MS diagnosis with clinical and radiological activity during the first years of MS treatment was observed. Assessment of NfLs by ELISA in CSF during the MS diagnostic process may be an available predictive marker of early clinical and radiological progression in MS patients.

Interleukin 6 (IL-6) promotes the production of factors that drive demyelination, playing a role in the development and progression of MS [21]. The release of IL-6 was markedly elevated in individuals with MS compared to those in the healthy control group [22]. In RRMS patients, those with impaired cognitive function, as reflected by their Mini Mental State Examination (MMSE) scores, exhibited notably increased IL-6 levels in peripheral blood [23]. In our study, increases in CSF levels of IL-6 was correlated with the risk of clinical progression assessed by EDSS and in patients with the clinical and/or radiological MS activity. Our results are consistent with the recently published results by Mathey et al. regarding the relationship between CSF IL-6 levels and the response to MS treatment in the first year [24].

CXCL13 is a small chemokine (10.3 kDa) that was first recognized in 1998 for its role in recruiting B lymphocytes into the CNS. It is also an important factor in the development of ectopic lymphoid follicles in the CNS in MS, which are linked to cortical demyelination, neuronal damage, and more severe disease progression [25]. CXCL13 production occurs early in MS, which suggests that the development of these structures may begin early in the disease, rather than only in later progressive stages. Therefore, the normal levels early on in disease stages may indicate a lower risk of neuroinflammatory events and a reduced likelihood of disability progression [25,26,27]. In patients with RRMS, levels of CSF CXCL13 were higher compared to those with PPMS. However, when focusing on patients without acute disease activity at baseline, these differences were no longer significant. This suggests that elevated CXCL13 is more likely reflective of acute disease activity rather than chronic progression in MS [9]. In MS patients, higher levels of CSF CXCL13 correlate with greater cortical thinning [28]. In individuals with MS, increased levels of CXCL13 are a strong indicator of upcoming disease activity, as reflected by MRI results and/or clinical relapses. Serum levels of CXCL13 were found to be lower in patients who later experienced disease activity in MS compared to those who did not [25]. In our study we noted a correlation of increased serum levels of CXCL13 with the need to change the first MS therapy, mainly due to ineffectiveness. This correlation was not observed when the only radiological or clinical activity group was analyzed. This is probably due to smaller group sizes, so research on the prognostic value of serum CXCL13 in MS should be continue.

The limitation of the study includes using ELISA in assessing NfLs instead of the SIMOA method, since we decided to use ELISA as the most accessible method in routine clinical practice.

The strength of the study is the homogeneous group of MS patients, and the serum and CSF biomarkers determined before the initiation of the first MS therapy. In particular, the levels of NfLs and IL-6 in the CSF may be a predictor of the disease course assessed as clinical and radiological activity. High levels of NfLs and IL-6 in the CSF of MS patients before therapy initiation may be an indication to use a highly effective therapy as the first treatment for MS.

## 4. Materials and Methods

This study was conducted prospectively and involved patients diagnosed with RRMS, who underwent the diagnostic process for MS at the Neurology Department of the Medical University of Lodz, Poland, between 2018 and 2021. The inclusion criteria targeted adult patients (aged 18 years or older) who had received an RRMS diagnosis according to the 2017 revised McDonald criteria. The mean time of observation was 60 months (median 50 months). Clinical relapse data, follow-up magnetic resonance imaging (MRI) results and information on disease-modifying therapies (DMTs) were collected from the hospital database. The neurological status of each patient was examined every 3 months and evaluated using the EDSS scale. Clinical activity was defined as occurrence of MS relapse and/or progression in EDSS score during observation period. MRI activity was defined as new T2 lesions and/or T1 Gd-enhancing lesions throughout the duration of the observation. The patients were subjected to a routine MRI of the brain performed on the 3T scanner (Vida, Siemens, Munich, Germany) at the beginning of the study and annually during the follow-up. The MRI protocol included the following sequences: a high-resolution axial 3-dimensional (3D) T1-weighted magnetization-prepared rapid gradient-echo (MPRAGE) (repetition time (TR) = 2200 ms, echo time (TE) = 2.46 ms, inversion time (TI) = 900 ms, field of view (FOV) = 256, number of slices = 167, and pixel size = 1 × 1 × 1 mm), sagittal isotropic 3D T2-weighted fluid-attenuated inversion recovery (FLAIR) (TR = 2560 ms, TE = 135 ms, TI = 6700 ms, FOV = 256, and number of slices = 192), proton density PD/T2-weighted (TR = 2560 ms, TE1/TE2 = 90/30 ms, FOV = 256, number of slices = 46, and slice thickness = 3.0 mm), and 3D T1-MPARAGE (repetition time (TR) = 2200 ms, echo time (TE) = 2.46 ms, inversion time (TI) = 900 ms, field of view (FOV) = 256, number of slices = 167, and pixel size = 1 × 1 × 1 mm) after intravenous contrast administration (gadolinium based, gadobutrol 0.1 mmol/kg body weight).

Serum and cerebrospinal fluid were obtained during diagnostic process, before treatment of MS was started. Concentrations of human IFN **γ**, IL-6, CHI3L1 (Biorbyt Ltd., Cambridge, UK), OPN, CXCL13, GFAP (Wuhan EIAab Science, Wuhan, China), and NfLs (Uman Diagnostics, Umea, Sweden) were measured in serum and CSF using appropriate ELISA kits. All reagents were used according to the manufacturer’s recommendations. Sera and CSFs were collected from all patients at the beginning of the study. To measure IFN-γ, IL-6, OPN, CXCL13, GFAP, NfLs, and CHI3L1, serum and CSF samples were added to plates coated with specific antibodies targeting the respective antigens. The target molecules in the samples adhered to the antibody-coated wells. After washing, biotinylated anti-human antibodies specific to the tested factors were introduced, followed by HRP-conjugated streptavidin and a TMB substrate solution. The intensity of the developed color was then detected at 450 nm EPOCH (BioTek, Agilent Technologies, Santa Clara, CA, USA) using a microplate reader. For intra-assay validation, four quality control (QC) samples were included on each plate. All assays were run in duplicates, maintaining an average coefficient of variation (CV) of 15% or less.

The study protocol was reviewed and approved by the Local Ethics Committee of the Medical University of Lodz (approval number /360/17/KE, 21 November 2017, RNN/231/18KE, 12 June 2018). Informed consent was obtained from all the subjects involved in the study.

## 5. Statistical Analysis

Statistical analysis was performed using Statistica 13 (StatSoft, Cracow, Poland). Non-parametric tests were employed due to violations of assumptions regarding the normality of data distribution and heterogeneity of variance, as revealed by the Shapiro–Wilk test and Levene’s test, respectively. Comparisons between dichotomous clinical variables (presence of clinical or radiological activity, worsening of EDSS score) were conducted using the Mann–Whitney U test. Bonferroni correction was used for *p*-value adjustment for multiple comparisons. Statistical significance was determined using a *p*-value of 0.05. After applying Bonferroni correction for seven multiple comparisons, the adjusted *p*-value was 0.007. Correlations between parameters were assessed using Spearman’s rank correlation coefficient.

## Figures and Tables

**Table 1:** Demographic characteristic.

Number of Participants	*n* = 121
Number of female/male (*n* (%))	81/40 (67.5%/32.5%)
Age (mean; years)	35.3
Time since first MS clinical presentation (mean; months)	36.1
Time of observation (mean; median; months)	60; 50
Baseline EDSS (mean)	1.5

**Table 2:** Correlation of serum and CSF biomarkers with clinical and radiological activity in MS patients.

Marker	RA (Z)	RA (*p*)	CP (Z)	CP (*p*)	RA and/or CP (Z)	RA and/or CP (*p*)
CHI3L1s	−0.472	0.464	1.346	0.179	0.904	0.368
CHI3L1 CSF	1.063	0.292	1.146	0.254	1.417	0.158
NfLs s	1.151	0.253	1.862	0.062	1.796	0.072
NfLs CSF	**2.020**	**0.042**	**2.812**	**0.004**	**2.809**	**0.004**
GFAP s	1.451	0.371	−0.482	0.777	0.517	0.759
GFAP CSF	−0.992	0.466	−0.404	0.764	−1.021	0.459
CXCL13 s	0.704	0.487	−0.580	0.565	0.240	0.811
CXCL13 CSF	0.579	0.569	−0.166	0.870	0.206	0.839
OPN s	−0.560	0.580	−0.185	0.854	−0.080	0.937
OPN CSF	0.106	0.917	1.911	0.055	1.287	0.199
IL-6 s	1.609	0.248	1.679	0.225	2.673	0.053
IL-6 CSF	1.708	0.119	**3.154**	**0.003**	**2.660**	**0.014**
INF s	−0.591	0.716	0.757	0.646	0.244	0.882
INF	−0.897	0.496	−0.546	0.672	−0.527	0.687

RA, radiological activity; CP, clinical progression; s, serum; CSF, cerebrospinal fluid; NfLs, neurofilament light chains.

## Data Availability

The original contributions presented in the study are included in the article. Further inquiries can be directed to the corresponding author.
